# Supplementary material for: Integrated molecular characterisation of the MAPK pathways in human cancers reveals pharmacologically vulnerable mutations and gene dependencies
Source: Commun Biol. 2021 Jan 4;4:9. doi: 10.1038/s42003-020-01552-6 (PMC7782843; doi:10.1038/s42003-020-01552-6)
Supplement: Supplementary file 2 — Supplementary Information [file 42003_2020_1552_MOESM2_ESM.docx]

# Supplemental Information Titles and Legends

**Supplementary Figure 1:** Complete connectivity of MAPK signalling pathway components. The nodes are coloured according to the MAPK pathway module (ERK1/2, p38, JNK or ERK5 pathways) in which the protein participates. The connectivity of network components was extracted from the BioGrid database^1^, Kinase Enrichment Analysis database^2^, Chromatin Enrichment Analysis database^3^, and UCSC super pathway databases.

**Supplementary Figure 2: (a)** Upset plot showing the mutual exclusivity and co-occurrence of mutations across the different MAPK pathway modules. Note that TP53 mutations are excluded from the plotted data. **(b)** Clustering of the 101 distinct cancer types based on the proportions of samples with mutations in each of the four MAPK signalling pathway modules (including TP53 mutations). Redder colour intensities denote higher percentages of mutations. The clustergram was produced using unsupervised hierarchical clustering with the cosine distance metric and complete linkage. The coloured bars on the heatmap show the overall frequency of gene mutations within the samples belonging to each cancer type represented within each column of the heatmap. **(** **(c)** Kaplan-Meier ^4^ curve of the disease-free survival periods of patients with tumours that have mutations to genes that encode the various classes of MAPK proteins. The numbers in parenthesis show the median OS or DFS periods. NaN (No a Number) represent undefined median OS or DFS period in that > 50% of patients survived beyond the study duration.


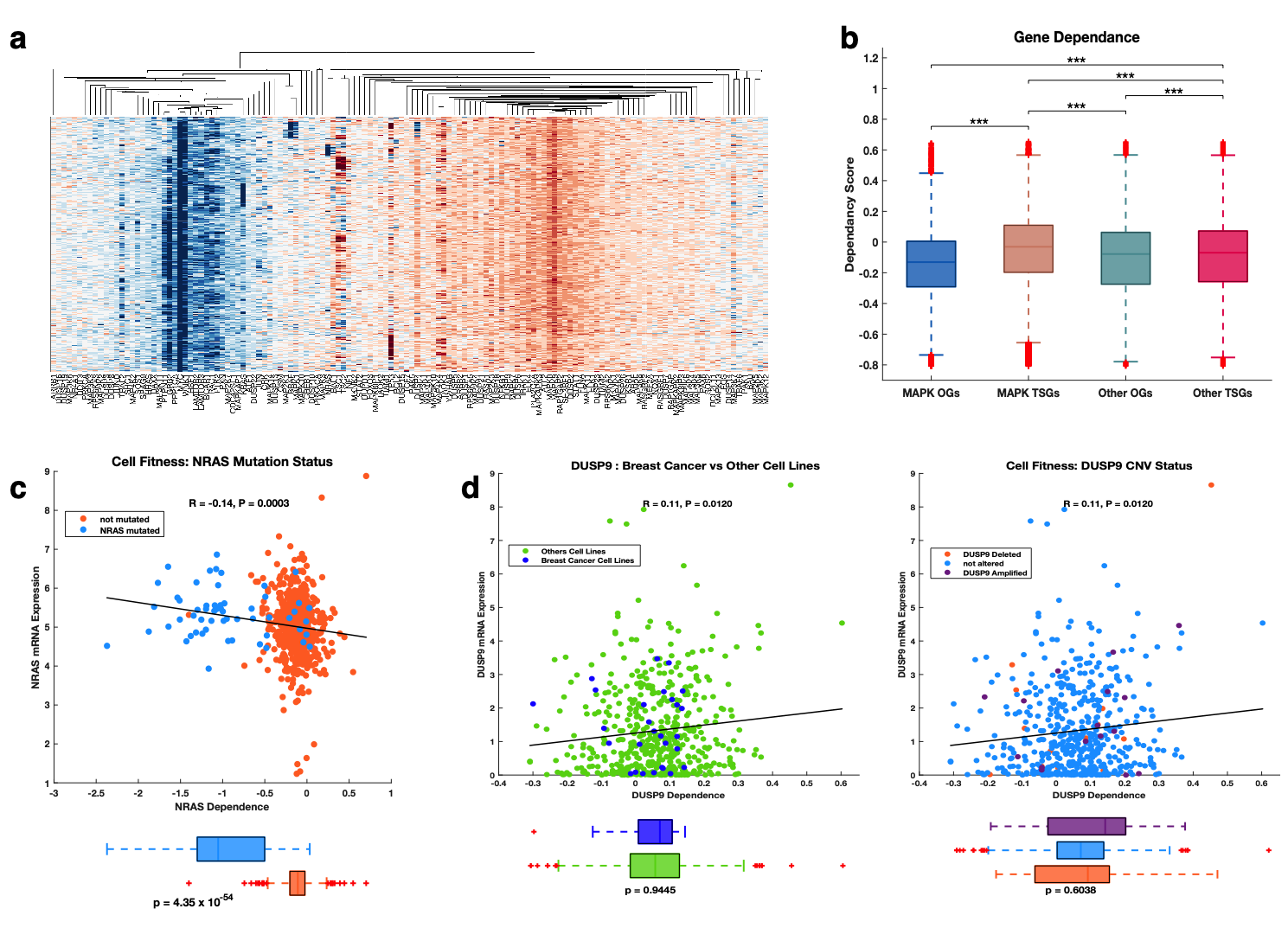


**Supplementary Figure 3: (a)** Clustergram of CRISPR-derived gene dependence scores across the cell lines. Bluer colours indicate reduced cell fitness and redder colours indicate increased cell fitness after CRISPR gene knockout. The clustergram was produced using unsupervised hierarchical clustering with the Euclidean distance metric and complete linkage. **(b)** Mean difference in the CRISPR-derived gene dependence scores between four categories of genes: (1) MAPK pathway genes that are oncogenes, (2) MAPK pathway genes that are tumour suppressor genes, (3) oncogenes that are not MAPK pathway genes, and (4) tumour suppressor genes that are not MAPK pathway genes. The CRISPR-derived gene dependence scores were compared between these groups using one-way analysis of variance. *** denotes statistical significance for adjusted p-values for each pairwise comparison using the Student’s *t*-test. On each box, the central mark indicates the median, and the bottom and top edges of the box indicate the 25th and 75th percentiles, respectively. The whiskers extend to the most extreme data points not considered outliers: we plotted the outliers individually using the '+' symbol. **(c)** Correlation and the mean difference in *NRAS* dependence scores between cell lines that harbour *NRAS* mutations and those that do not harbour *NRAS* mutations. **(f)** Correlation between *DUSP9* mRNA transcript levels and *DUSP9* dependence scores, and the mean difference in *DUSP9* dependence scores between breast cancer (the cancer type with most *DUSP9* mutations) cell lines and all other cancer cell lines. **(g)** Correlation between *DUSP9* mRNA transcript levels and *DUSP9* dependence scores and the mean difference in *DUSP9* dependence between the cell lines that gained copies of the *DUSP9* gene, those that have lost a copy of the *DUSP9 gene*, and those with normal copies of the *DUSP9* gene.

**Supplementary Figure 4:** Mean difference comparisons between the pooled CRISPR-derived gene dependence scores of each cancer type for a particular MAPK signalling pathway module vs the pooled CRISPR-derived gene dependence scores of all the genes that are not MAPK pathway genes for all 688 of the cancer cell lines (the green coloured boxplots). The blue boxplots indicate statistically significant losses of cell fitness and the red boxplots statistically significant increases in cell fitness. P-values for each comparison were calculated using Welch's t-test. On each box, the central mark indicates the median, and the left and right edges of the box indicate the 25th and 75th percentiles, respectively. The whiskers extend to the most extreme data points not considered outliers, and the outliers are plotted individually using the '+' symbol.

**Supplementary Figure 5:**  Mean difference comparisons between **(a)** the pooled CRISPR-derived gene dependence scores of each cancer type for genes that encode classes of MAPK pathway proteins versus the pooled Achilles dependence scores for those genes across all 688 of the cancer cell lines (the green coloured boxplots). **(b)** The pooled CRISPR-derived gene dependence scores of each cancer type for genes that encode the classes of MAPK pathway protein versus the pooled CRISPR-derived gene dependence scores of all the genes that are not MAPK pathway genes across all 688 of the cell lines. Note that we made all comparisons with the pooled dependence score across all 688 of the cell lines (the green coloured boxplots). See Supplementary Figure 5 for description of the boxplots.


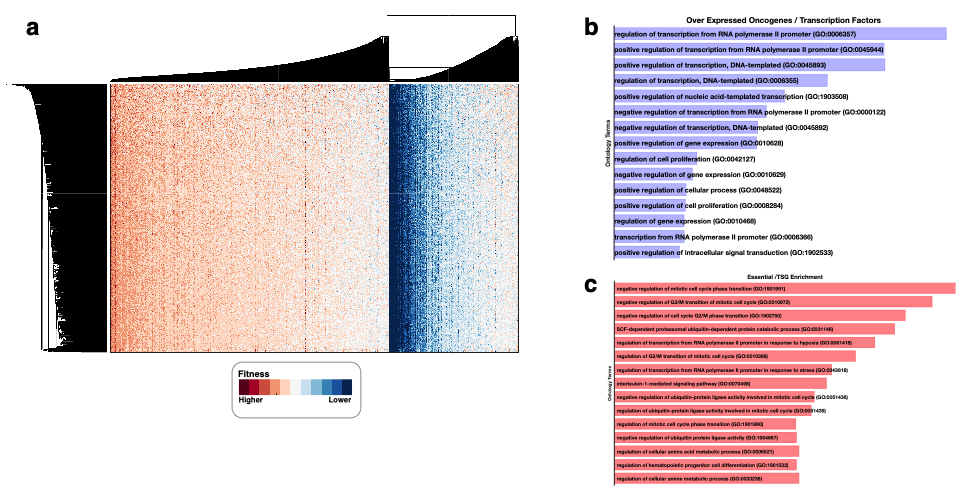


**Supplementary Figure 6: (a)** Clustering of the CRISPR-derived gene dependence scores across the 18023 genes that were profiled by the Achilles project. Bluer colour indicate reduced fitness, whereas redder colours indicate increased fitness of the cell lines after CRISPR genes knockouts. **(b)** Showing the top-ranked gene ontology biological process terms enriched for gene sets that showed a negative linear Pearson's correlation coefficient < -0.3. The genes list is composed of mostly oncogenes and transcription factors such as the *MYC* proto-oncogene (see Figure 5b). Also, see Supplementary File 3 for the complete list of gene ontology biological process terms that we found enriched. **(c)** Showing the top-ranked gene ontology biological process terms enriched for the gene sets that showed a positive linear Pearson’s correlation coefficient > 0.3. The gene list is composed of mostly tumour suppressor genes such as *CDKN2A*. Also, see Supplementary File 3 for the complete list of gene ontology biological process terms that we found enriched.


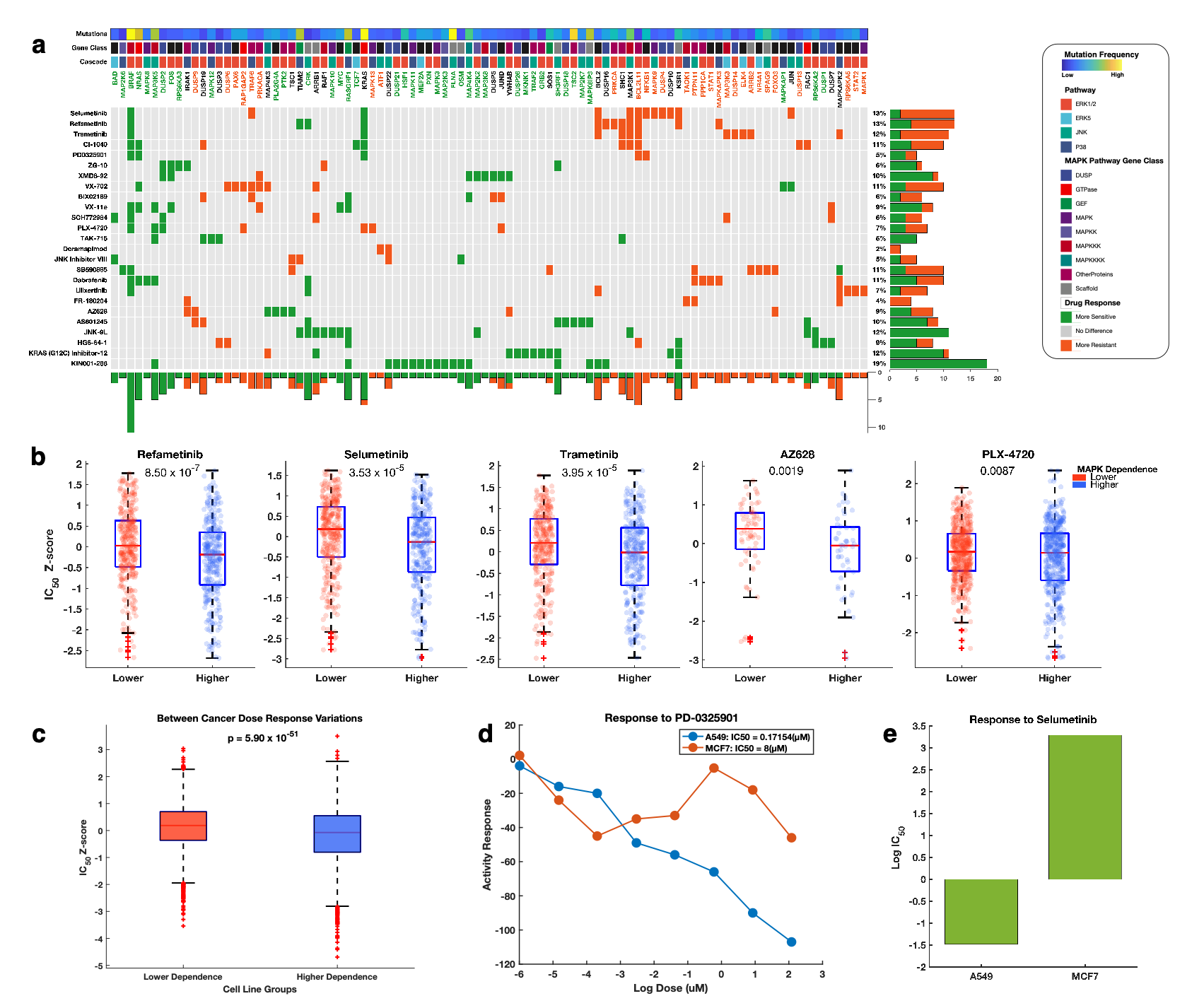


**Supplementary Figure 7: (a)** This integrated plot shows the relationship between gene mutations and the drug responses of the cancer cell lines. From top to bottom, the panel indicates: Mutations; the overall mutation frequencies observed for the gene along that column. Gene Class; the class of the MAPK pathway protein encoded by the gene. Pathway Module; the MAPK pathway module in which the gene participates. Heatmap; The marks on the heatmap are coloured based on how mutations in the gene in each column affect the efficacy of the drug given along each row: (1) with green denoting significantly increased sensitivity between cell lines that have mutations and those that do not have mutations in the gene, (2) grey for no statistically significant difference, and (3) orange denoting significantly increased resistance. The gene (column labels) are coloured based on the overall calculated effect of mutations on the efficacy of the drug given along each row: green; all the cell lines are significantly more sensitive to all the MAPK pathway inhibitors, orange; all the cell lines are significantly more resistant to all the MAPK pathway inhibitors, and black; a mixed response to MAPK pathway inhibitors. The bar graphs represent the total numbers of drugs with dose-responses that are significantly increased (green) or decreased (orange). **(b)** Comparison of the dose-response profiles to MAPK inhibitors between the cancer cell lines with higher dependence (boxplots with blue scatter point) on MAPK signalling and those with lower dependence (boxplots with red scatter point) on MAPK signalling. The cancer cell lines that have either a higher or lower dependency on MAPK signalling are given Supplementary File 4. Boxplots show the logarithm transformed mean IC50 values of the cancer cell lines of each group. Note that only the boxplots for the statistically significant comparisons are shown. On each box, the central red mark indicates the median, and the bottom edge represents the 25th percentiles, whereas the top edge of the box represents 75th percentiles. The whiskers extend to the most extreme data points not considered outliers, and the outliers are plotted individually using the ‘ + ‘ symbol. The scatter point within each box plot show the overall distribution of the data points. The scatter point within each box plot show the overall distribution of the data points.

**(c)** Overall comparison of the dose-responses to MAPK inhibitors between the cell lines with higher dependence on MAPK signalling and those with lower dependence on MAPK signalling. **(d)** Dose-response of the A549 and MCF7 cell lines to PD-0325901 as profiled by the GDSC. (e) Dose-response of the A549 and MCF7 cell lines to selumetinib as profiled by the CCLE.

**Reference**

1. Oughtred, R. *et al.* The BioGRID interaction database: 2019 update. *Nucleic Acids Res.* **47**, D529–D541 (2019).

2. Lachmann, A. & Ma’ayan, A. KEA: kinase enrichment analysis. *Bioinformatics* **25**, 684–6 (2009).

3. Lachmann, A. *et al.* ChEA: transcription factor regulation inferred from integrating genome-wide ChIP-X experiments. *Bioinformatics* **26**, 2438–2444 (2010).

4. Goel, M. K., Khanna, P. & Kishore, J. Understanding survival analysis: Kaplan-Meier estimate. *Int. J. Ayurveda Res.* **1**, 274–8 (2010).
