# Supplementary material for: Integrated molecular characterisation of the MAPK pathways in human cancers reveals pharmacologically vulnerable mutations and gene dependencies
Source: Commun Biol. 2021 Jan 4;4:9. doi: 10.1038/s42003-020-01552-6 (PMC7782843; doi:10.1038/s42003-020-01552-6)
Supplement: Supplementary file 8 — Reporting Summary [file 42003_2020_1552_MOESM8_ESM.pdf]

nature research

Corresponding author(s): Musalula Sinkala

Last updated by author(s): Nov 15, 2020

Reporting Summary

Nature Research wishes to improve the reproducibility of the work that we publish. This form provides structure for consistency and transparency in reporting. For further information on Nature Research policies, see our Editorial Policies and the Editorial Policy Checklist.

Statistics

For all statistical analyses, confirm that the following items are present in the figure legend, table legend, main text, or Methods section.

n/a	Confirmed	
☐	☒	The exact sample size (*n*) for each experimental group/condition, given as a discrete number and unit of measurement
☒	☐	A statement on whether measurements were taken from distinct samples or whether the same sample was measured repeatedly
☐	☒	The statistical test(s) used AND whether they are one- or two-sided<br>*Only common tests should be described solely by name; describe more complex techniques in the Methods section.*
☐	☒	A description of all covariates tested
☐	☒	A description of any assumptions or corrections, such as tests of normality and adjustment for multiple comparisons
☐	☒	A full description of the statistical parameters including central tendency (e.g. means) or other basic estimates (e.g. regression coefficient) AND variation (e.g. standard deviation) or associated estimates of uncertainty (e.g. confidence intervals)
☐	☒	For null hypothesis testing, the test statistic (e.g. F, t, r) with confidence intervals, effect sizes, degrees of freedom and P value noted<br>*Give P values as exact values whenever suitable.*
☒	☐	For Bayesian analysis, information on the choice of priors and Markov chain Monte Carlo settings
☒	☐	For hierarchical and complex designs, identification of the appropriate level for tests and full reporting of outcomes
☐	☒	Estimates of effect sizes (e.g. Cohen's *d*, Pearson's *r*), indicating how they were calculated

Our web collection on statistics for biologists contains articles on many of the points above.

Software and code

Policy information about availability of computer code

Data collection	Data were retrieved from the various repertoires using Application Program Interfaces provided by the cBioPortal, International Cancer Genome Consortium, Genomics of Drug Sensitivity in Cancer and among others.
Data analysis	All analyses were performed in MATLAB 2019b. Custom code written in MATLAB for processing and analysis of the data presented here is freely available at https://github.com/smsinks/Integrated-Analysis-of-MAPK-Pathway-Across-Human-Cancers. The repository includes some pre-downloaded datasets and conversion files required for the analysis.

For manuscripts utilizing custom algorithms or software that are central to the research but not yet described in published literature, software must be made available to editors and reviewers. We strongly encourage code deposition in a community repository (e.g. GitHub). See the Nature Research guidelines for submitting code & software for further information.

Data

Policy information about availability of data

All manuscripts must include a data availability statement. This statement should provide the following information, where applicable:

- Accession codes, unique identifiers, or web links for publicly available datasets
- A list of figures that have associated raw data
- A description of any restrictions on data availability

The data that support our results are available from the following repositories: cBioPortal; https://www.cbioportal.org/, the Genomics of Drug Sensitivity in Cancer; https://www.cancerrxgene.org/, the Cancer Cell Line Encyclopedia; https://portals.broadinstitute.org/ccle/data, the LINCS project; http://www.lincsproject.org, the Genotype-Tissue Expression project; https://gtexportal.org/home/, the COSMIC Consensus Cancer Genes; https://cancer.sanger.ac.uk/census, and the Project Achilles; https://depmap.org/portal/.

April 2020

Field-specific reporting

Please select the one below that is the best fit for your research. If you are not sure, read the appropriate sections before making your selection.

- [x] Life sciences
- [ ] Behavioural & social sciences
- [ ] Ecological, evolutionary & environmental sciences

For a reference copy of the document with all sections, see nature.com/documents/nr-reporting-summary-flat.pdf

Life sciences study design

All studies must disclose on these points even when the disclosure is negative.

Sample size	We did not calculate the sample size for our study. We retrieved all available data of 40,848 patients afflicted by 101 different human cancers and the CRISPR dependency scores, mRNA transcription level, and gene mutations data of over 700 cancer cell lines. We are confident that such a large sample size is sufficient to answer the various questions that we addressed.
Data exclusions	No data were excluded from the analyses.
Replication	Our analyses were based on secondary data; therefore, no experimental were done by us.
Randomization	No randomization was performed as our study was based on the analysis of secondary data.
Blinding	No blinding was necessary as our study involved the analysis of clinical and molecular data of various tumours and cancer cell lines.

Reporting for specific materials, systems and methods

We require information from authors about some types of materials, experimental systems and methods used in many studies. Here, indicate whether each material, system or method listed is relevant to your study. If you are not sure if a list item applies to your research, read the appropriate section before selecting a response.

Materials & experimental systems

n/a	Involved in the study	
[x]	[ ]	Antibodies
[x]	[ ]	Eukaryotic cell lines
[x]	[ ]	Palaeontology and archaeology
[x]	[ ]	Animals and other organisms
[x]	[ ]	Human research participants
[x]	[ ]	Clinical data
[x]	[ ]	Dual use research of concern

Methods

n/a	Involved in the study	
[x]	[ ]	ChIP-seq
[x]	[ ]	Flow cytometry
[x]	[ ]	MRI-based neuroimaging
